# Bibliography

**Published:** 1892-10

**Authors:** 


### Bibliography.

FIVE HUNDRED AND SIXTY-SEVEN USEFUL HINTS FOR THE BUSY DENTIST. By William H. Steele, D.D.S. Published by the Wilmington Dental Manufacturing Company, Philadelphia, 1892.

The compilation of such a book as this means care and labor of no ordinary amount. The author gives as the reason for his effort in this direction that "for many years I have felt the need of a little hand-book such as I have attempted. . . . My intention has been to furnish an answer as accurately and with sufficient detail, in as compact and accessible a form as possible, to all questions and difficulties that may arise in our every-day life, at the chair or bench."

The book is divided into five departments,—A, B, C, D, E. Department A is devoted to hints in operating; B, to mechanical work; C, to crown- and bridge-work; D, to a variety of useful suggestions in many departments of dental work; E, to a miscellaneous collection of hints of more or less value to the practical man.

For the service this book is intended to perform it is well prepared, and will, no doubt, be of great value to the beginner in practice, but to those thoroughly educated a collection of this kind should not be needed. The trained dentist ought to have all the minor points of every-day practice thoroughly absorbed and capable of being brought into use at a moment's notice. This class, it may be feared, is not a large one, hence a book of this kind will have a special value to a certain proportion of men who will always require a constant stimulus to memory.

The Medical and Dental Register, Directory, and Intelligencer of Pennsylvania, New Jersey, and Delaware. George Kiel, publisher: Philadelphia, 1892.

This book contains a complete list of the National and State Medical and Dental Associations, with their officers and date of meetings, Medical and Dental Colleges of the United States, and other very valuable material, Medical and Dental Laws, Hospitals, Homes, etc., etc., also the lists of Medical and Dental practitioners, with their school and year of graduation, post-office addresses, and office hours.

Directories, to be of any value, must be carefully compiled. This seems to be arranged with a conscientious effort in this direction. Mistakes in names and residences are perhaps unavoidable, but this, as far as we are able to determine, is exceptionally free from these. From the comparatively limited area of country covered, this is to be expected.

The additional matter connected with it makes it extremely valuable for reference in the special field for which it was designed.

